# LHPP suppresses gastric cancer progression via the PI3K/AKT/mTOR signaling pathway

**DOI:** 10.7150/jca.78098

**Published:** 2022-10-31

**Authors:** Danfang Wang, Jianhui Li, Wenhan Li

**Affiliations:** 1 Department of Oncology, Xi'an Gaoxin Hospital, Xi'an, Shaanxi, China.; 2 Department of Surgical Oncology, Shaanxi Provincial People's Hospital, Xi'an, Shaanxi, China.; 3 The Third Affiliated Hospital, School of Medicine, Xi'an Jiaotong University, Xi'an, Shaanxi, China.

**Keywords:** LHPP, Gastric cancer, EMT, the PI3K/AKT/mTOR pathway.

## Abstract

Emerging evidence has revealed the anti-oncogenic role of LHPP in several malignancies. The current study aims to explore the underlying mechanism of LHPP in gastric cancer (GC). We used the TCGA and GEO databases to investigate the expression profile, prognostic value, and cellular function of LHPP in GC. LHPP expression pattern were further verified using clinical samples by immunohistochemistry and western blot analysis. Moreover, stable cancer cell lines with LHPP overexpression or knockdown were established. CCK-8 assay, colony formation assay, transwell assay, qRT-PCR, and western blot analysis were performed to uncover the underlying mechanism concerning LHPP during the progression of GC. The present study revealed that LHPP was down-regulated in GC cell lines and tissue samples at both mRNA and protein level. LHPP inhibited GC cells proliferation, migration, invasion, and epithelial-mesenchymal transition (EMT) *in vitro*. Mechanically, LHPP overexpression led to decreased level of PI3K/AKT/mTOR pathway phosphorylation, while LHPP depletion produced opposite results. Moreover, our data indicated that the enzymatic active site of LHPP is neither the cysteine residue at position 226 nor at position 53 in GC. Overall, our study demonstrated that LHPP function as a tumor suppressor gene in GC by regulating the PI3K/AKT/mTOR pathway.

## Introduction

Gastric cancer (GC) is one of the most common malignancies worldwide, accounting for 768,793 deaths in 2020 1. Although therapeutic strategies have been improved, the treatment efficacy remains limited. Hence, investigation of the molecular mechanisms in GC progression and identification of the potential therapeutic targets are urgently needed in the current scientific research.

Phospholysine phosphohistidine inorganic pyrophosphate phosphatase (LHPP) gene is located on chromosome 10q26.13, which encodes a highly evolutionarily conserved histidine phosphatase 2. LHPP was considered as a genetic marker of alcohol dependence and major depression 3-5. In 2018, Sravanth et al.[6]first confirmed the antitumor effect of LHPP in hepatocellular carcinoma. Subsequent researches with cell- or animal-based evidence support the correlation between LHPP and tumorgenesis had been widely conducted. LHPP was found to be down-regulated and associated with cancer progression and favorable prognosis in many cancers 7-9. Our research group also reported functional links between LHPP and tumorigenesis in colorectal cancer 10, 11. In addition, we recently conducted a comprehensive bioinformatics analysis of LHPP gene function in pan-cancer by using the Cancer Genome Atlas (TCGA) database, the GTEx database, and the Clinical Proteomic Tumor Analysis Consortium (CPTAC) database. In the current study, we focused on exploring the expression profile and potential mechanism of LHPP in GC. Lin et al. 12 recently published an article claims that LHPP is regulated by m6A methylation and regulates the metabolism of GC by changing the acetylation level. However, based on our bioinformatics analysis and preliminary data, we found that LHPP may suppress cell proliferation of GC via down-regulating p‑PI3K/p‑AKT expression levels. This discrepancy may due to multiple signaling pathways involved in regulating the biological functions of LHPP in GC cells.

Our study, for the first time, we used the TCGA project and GEO databases to evaluate the expression pattern and prognosis role of LHPP in GC. Moreover, cell function, epithelial-mesenchymal transition (EMT), histidine phosphorylation, and the PI3K/AKT/mTOR pathway alteration were investigated to further uncover the underlying mechanism of LHPP in GC progression.

## Methods

### Data Sources and Preparation

The mRNA expression profile as well as clinicopathological parameters of 407 GC patients were downloaded from The Cancer Genome Atlas (TCGA) database (https://portal.gdc.cancer.gov/). In addition, GSE84437 microarray series were downloaded from the Gene Expression Omnibus dataset (GEO) at the NCBI.

### Patients and clinical specimens

The study material consisted of 52 tumor tissue samples, paired para-cancerous histological normal tissues (PCHNTs) which are obtained during curative surgery. The inclusion criteria were as follows: (1) histological confirmed GC; (2) complete clinicopathological and follow-up data. The exclusion criteria were as follows: (1) histological diagnosed second primary tumor; (2) history of gastric resection or preoperative chemotherapy/radiation therapy. All samples were immediately frozen and stored at -80℃. All patients voluntarily joined this study with written informed consent to have their biological specimens analyzed. This study was announced by the Ethical Committee of the Shaanxi Provincial People's Hospital (reference number: 2021-014).

### Immunohistochemistry (IHC)

Immunohistochemistry was performed as previously described 11. Briefly, GC tissue and PCHNTs samples were embedded in paraffin, and 4-µm-thick sections were dewaxed in xylene and were autoclaved in citrate buffer. After blocking non‑specific staining with goat serum, primary rabbit polyclonal antibodies for LHPP (dilution 1:200, catalog no.15759‑1‑AP, Proteintech) were incubated with tissue sections overnight at 4˚C. LHPP expression was evaluated by using IHC scores. Each field was scored independently by two pathologists.

### Cell culture and transfection

We used four GC cell lines (AGS, SNU-1, HGC-27, and NCI-N87) and one gastric mucosal epithelial cell line (GES-1) for subsequent analysis. Those cell lines were culture in strict accordance with their respective protocols. We also construct lentiviruses containing overexpression vectors of LHPP or expressing shRNA targeting LHPP for cell function analysis. Those lentiviruses were bought from GeneChem, Shanghai. The shLHPP sequences are as follows, sh-228: CTGTGCTCATATCACTGGGAA, sh-229: CAGCTTCAGAGGCTGGGATTT, sh-230: TGCCAGATCCTGAAGGAGCAA.

### Real-time quantitative PCR assay

We used Fastagen 200 kit (Shanghai, China) and TRIzol reagent (Ambion, life technologies, USA) to extract RNA according to their respective instructions. After synthesizing cDNA with Primescript RT reagent kit (TaKaRa) and mixing appropriate volume of cDNA with SYBR Premix Ex Taq^TM^ II (Tli RNaseH Plus) (TaKaRa) reagents, samples were tested by CFX96 Real-Time PCR Detection System (Bio-Rad, California, USA). The following primers were used: GAPDH: forward: 5-CACCCACTCCTCCACCTTTGA-3, reverse: 5-TCTCTCTTCCTCTTGTGCTCTTGC-3. LHPP: forward: 5'‑GCTTCAGAGGCTGGGATTTGAC‑3', reverse, 5'‑AATTACCACACAGTTTGGGTTGGA ‑3'. All reactions were performed in triplicate.

### Western blot analysis

The western blot analysis procedure was conducted as previously reported^11^. The primary or secondary antibodies and their respective diluted concentrations we used in this study are as follows: (LHPP: dilution 1:200, catalog no. 15759‑1‑AP, Proteintech; GAPDH, dilution 1:2000, catalog no. 60004‑1‑AP, Proteintech; β-actin, dilution 1:2000, catalog no. 20536‑1‑AP, Proteintech; AKT, dilution 1:500, catalog no. 10176‑2‑AP, Proteintech; p‑AKT, dilution 1:1000, catalog no. 4060S, CST; PI3K, dilution 1:1000, catalog no. 4249S, CST; p-PI3K, dilution 1:1000, catalog no. ab278545, abcam; E-cadherin, dilution 1:1000, catalog no. 9782T, CST; β-catenin, dilution 1:1000, catalog no. 9782T, CST; N-cadherin, dilution 1:1000, catalog no. 9782T, CST; Vimentin, dilution 1:1000, catalog no. 9782T, CST; Snail , dilution 1:1000, catalog no. 9782T, CST; Slug, dilution 1:1000, catalog no. 9782T, CST; ZEB1, dilution 1:1000, catalog no. 9782T, CST; mTOR, dilution 1:1000, catalog no. 9862T, CST; p-mTOR, dilution 1:1000, catalog no. 9862T, CST; p-pS6k(C371), dilution 1:1000, catalog no. 9862T, CST; p-pS6k(T389), dilution 1:1000, catalog no. 9862T, CST; 1-Histidine phosphorylation (1- PHis), dilution 1:1000, catalog no. MABS1330, Merk; 3-PHis, dilution 1:1000, catalog no. MABS1352, Merk); secondary antibody (dilution 1:10000, catalog no. SA00001-2/ SA00001-1, Proteintech).

### Cell functional assays

We measured cell viability, cell migration and invasion by Cell Counting Kit-8 (CCK-8) assay, colony formation analysis and transwell analysis according to their respective protocols. The detailed information of experimental procedure can be found in our previously published articles 11.

### Statistical analysis

We used SPSS 13.0 software (SPSS, Chicago, IL, USA), GraphPad Prism 5.0 (GraphPad Software, La Jolla, CA), and R software version 3.6.1 (http://www.r-project.org) for data processing. The t-test, the one-way analysis of variance, and the χ2 test were performed with their corresponding application scenario. *P* < 0.05 was considered statistically significant.

## Results

### LHPP is down-regulated and may correlate with poor prognosis in GC

**Figure 1:**
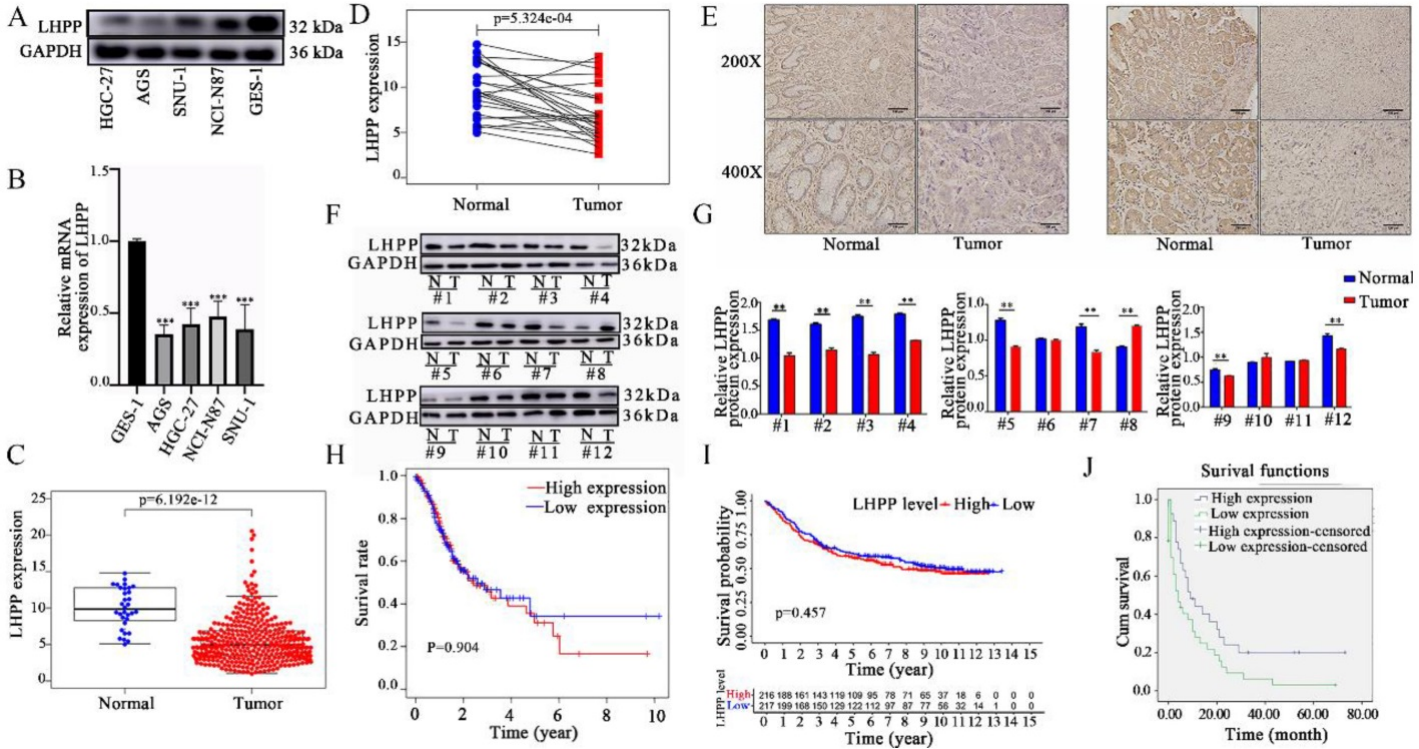
Relative protein (A) and mRNA (B) expression level of LHPP in four human gastric cancer (GC) cell lines (HGC-27, AGS, SNU-1, and NCI-N87) and one gastric epithelial cell line (GES-1). (***P < 0.001). (C) Comparison of LHPP mRNA expression in GC and non-cancerous tissues in the TGCA database. (D) Comparison of LHPP mRNA expression in 54 paired gastric and adjacent non-cancerous tissues in the TCGA database. (E) Immunohistochemistry analysis of LHPP in GC and adjacent normal tissues. Scale bar, 150 μm. (F) GC and normal tissues were subjected to Western blot analysis of LHPP. GAPDH serves as internal control. (G) Relative protein expression levels of LHPP were calculated using GraphPad Prism 6 software. N, normal tissues; T, tumor tissues. *P < 0.05, **P < 0.01. (H-J) Kaplan-Meier survival analysis of the association between LHPP expression and overall survival in GC recruited from the TCGA database, the GEO database, and the Shaanxi Provincial People's Hospital, respectively.

The expression levels of LHPP in four GC cell lines (HGC-27, AGS, SNU-1, and NCI-N87) and one gastric epithelial cell line (GES-1) were analyzed via qRT-PCR and western blotting analysis. Figure 1A and 1B demonstrated that compared with gastric epithelial cell lines, LHPP expression was significantly lower in all four GC cell lines in both transcription and translation level (All *P* < 0.05). Among them, LHPP protein expression was highest in NCI-N87 cells while lowest in AGS cells.

Next, the mRNA expression level of LHPP between tumor and normal tissues was further verified using the TCGA database. First, we obtained the RNA sequencing data of 407 cases (including 32 normal tissues and 375 tumor tissues) from the TCGA database. As shown in Figure 1C, the expression level of LHPP was significantly down-regulated in GC tissues than that in normal controls (*p* < 0.05). In particular, the comparison result of LHPP expression in paired-sample analysis further demonstrated that LHPP is definitely down-regulated in tumor than in the matched noncancerous samples (*p* < 0.05, Figure 1D). Furthermore, we did not detect significant correlation between the expression level with age, gender, and TNM stage (Supplementary Figure S1). Last but not least, we subsequently recruited 52 paired tissue samples from our center into this study. Both western blot and IHC score results demonstrated that the LHPP protein expression level was down-regulated in GC relative to normal controls (Figure 1E-G). Further analysis revealed that the correlation between LHPP expression and clinicopathological parameters is insignificant (All *p* > 0.05, Table 1).

Finally, Kaplan-Meier survival analysis was applied to clarify the relationship between LHPP expression and GC prognosis. For the data from the TCGA and GEO databases, we divided our research subjects into high-expression and low-expression groups based on the median mRNA expression level of LHPP. As shown in Figure 1H-I, no significant difference in the OS was observed between patients with high and low expression of LHPP in both of those two datasets. For the data from our medical center, we defined LHPP protein is extensively expressed when its IHC score reaches 3 points or more. The results showed that even though the baseline information of the patients between online dataset and our research center was similar (Supplementary Table S1), follow-up data from our center showed that patients with higher expression levels of LHPP exhibited extended OS (Figure 1J).

### LHPP suppresses cell proliferation and colony formation *in vitro*

**Figure 2:**
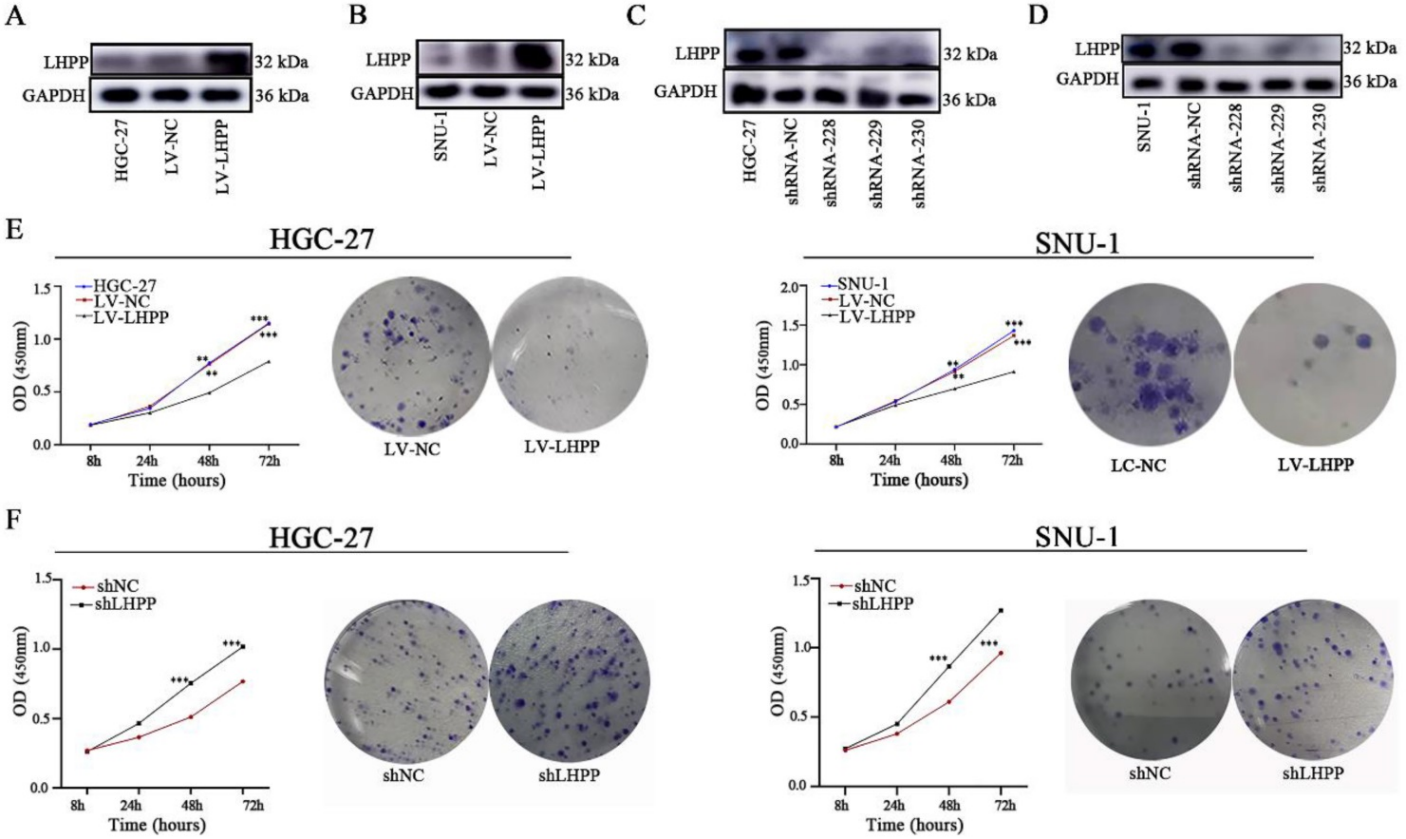
HGC-27 and SNU-1 cell lines with stable LHPP overexpression (A-B) or knockdown (C-D) were generated and confirmed by western blot analysis. (E) Effects of LHPP up-regulation on the proliferation and colony formation in HGC-27 and SNU-1 cell lines. ***P < 0.001. (F) Effects of LHPP down-regulation on the proliferation and colony formation in HGC-27 and SNU-1 cell lines. ***P < 0.001.

We used two cell lines (SNU-1 and HGC-27) with moderate LHPP expression levels for LHPP silencing and overexpression construction. GC cells were infected with lentivirus-shLHPP or LHPP lentiviruses and their corresponding negative controls strictly according to the protocol. Western blot analysis demonstrated LHPP expression in shRNA-228, shRNA-229 and shRNA-230 groups were all obviously lower than in shRNA-NC group, whereas overexpressed in LV-LHPP group (Figure 2A-D).

We next conducted CCK8 and colony formation assays to measure cell viability. Results demonstrated that overexpressed LHPP significantly suppresses cell growth in the HGC-27 and SNU-1 cell lines compared with their respective control cells (*p* < 0.05, Figure 2E). Conversely, LHPP knockdown increased the proliferation of those two GC cell lines (*p* < 0.05, Figure 2F). Likewise, clonal formation capability was greatly reduced in the LHPP-overexpressing group while significantly increased in the LHPP-knockdown group when comparing with their respective control groups, suggesting that LHPP indeed negatively regulates GC cell proliferation *in vitro* (Figure 2E-F).

### LHPP suppresses cell migration, invasion, and EMT process *in vitro*

**Figure 3:**
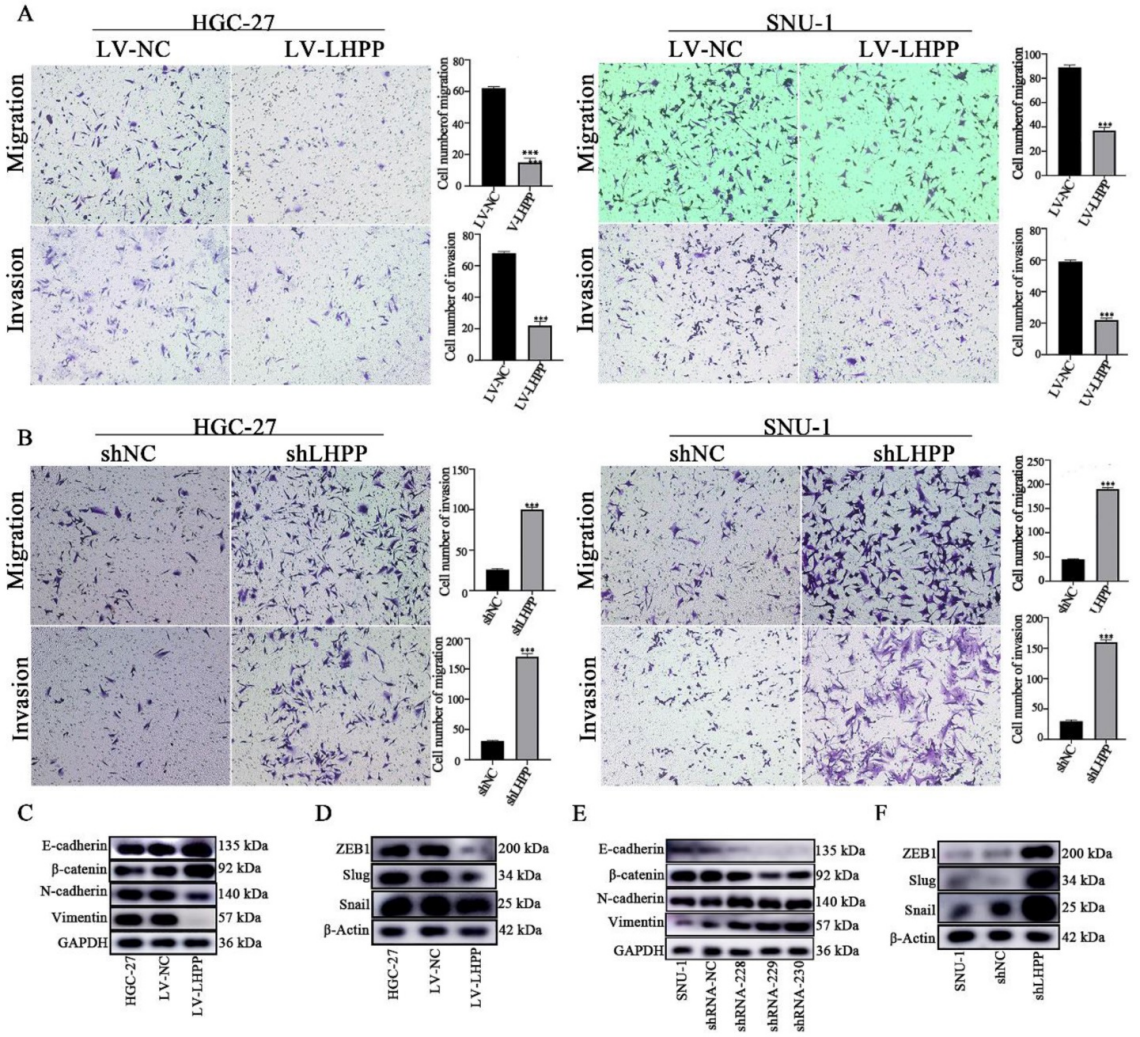
Effect of LHPP overexpression (A) or knockdown (B) on the migration and invasion of HGC-27 and SNU-1 cell lines compared to their corresponding control groups. ***P < 0.001. (C-F) EMT-related proteins including E-cadherin, β-catenin, N-cadherin, Vimentin, ZEB1, Snail, and Slug were tested using Western blot analysis in HGC-27 and SNU-1 cell lines with stable overexpression or knockdown of LHPP.

Transwell assays were conducted to investigate the migration and invasion status of LHPP in GC cells. Figure 3A-B showed that LHPP overexpression in GC cells inhibited migration, whereas LHPP knockdown promoted migration. The transwell invasion assay showed the same trend as migration assay above, demonstrating LHPP inhibited cell migration and invasion ability.

Epithelial-mesenchymal transition (EMT) is considered to be one of the key events related to tumor progression. We next explored the impact of LHPP expression level on EMT-associated molecules in GC cell lines. As illustrated in Figure 3C-D, increasing LHPP expression significantly inhibited N-cadherin, Vimentin, Snail, Slug and ZEB1, while induced E-cadherin and β-catenin levels. As expected, LHPP depletion produced opposite results. (Figure 3E-F). These results indicated that LHPP could repress cell migration, invasion, and EMT process in GC.

### LHPP suppresses the malignant behavior of GC cells via activating the PI3K/AKT/mTOR pathway

**Figure 4:**
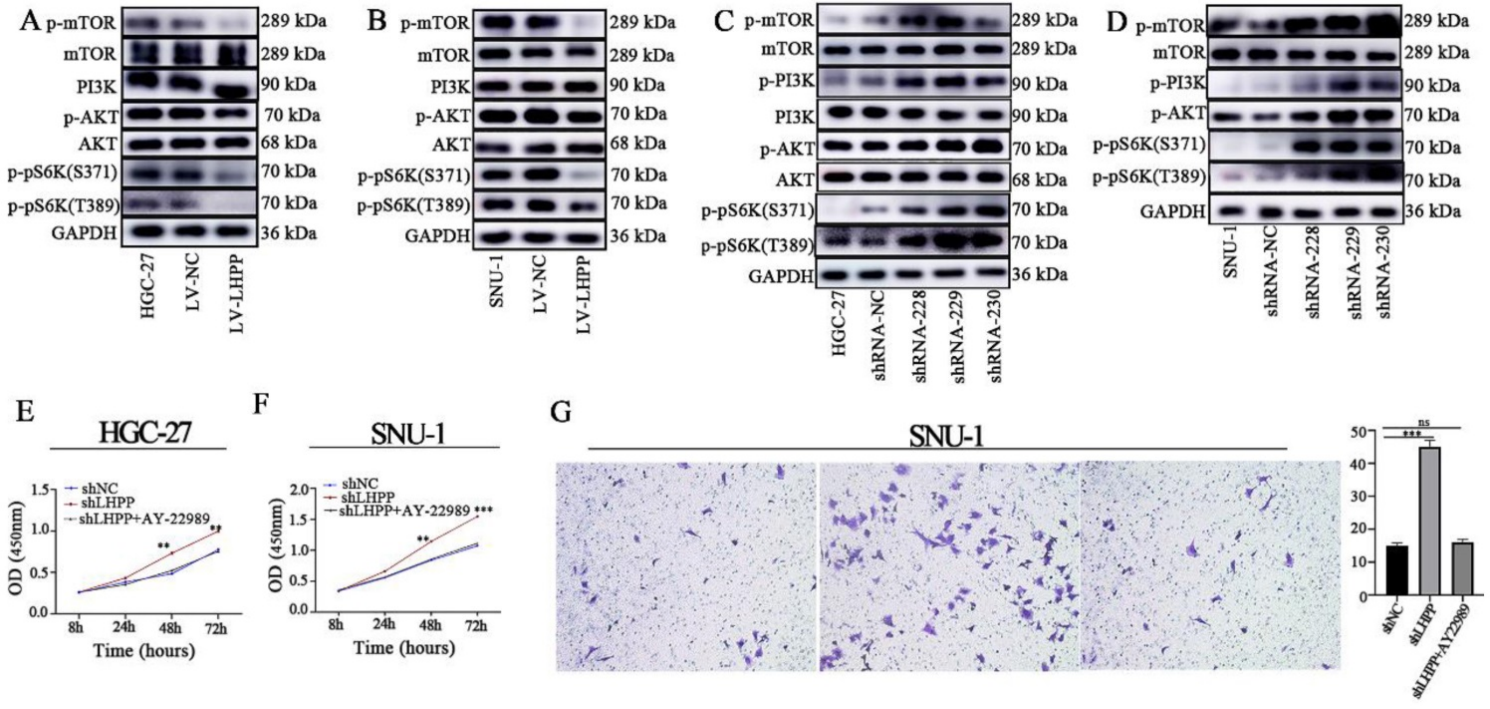
Pivotal molecules of PI3K/AKT/mTOR signaling pathway, including AKT, p-AKT, mTOR, p-mTOR, PI3K, p-pS6K (S371), and p-pS6K (T389), were evaluated HGC-27 (A, C) and SNU-1 (B, D) cell lines with stable overexpression or knockdown of LHPP. The CCK-8 assay (E-F) and transwell assay (G) was used for evaluating the influence of AY-22989 on cell proliferation and migration abilities in HGC-27 and SNU-1 cell lines with stable overexpression or knockdown of LHPP.

Research has shown that the AKT/mTOR, NF-κB, and ERK/MAPK signaling pathways are closely related to tumor EMT 13, 14. Besides, LHPP has been proven to have a strong connection with PI3K/AKT 7, 9, 15, 16. Thus, we examined whether LHPP affects biological behavior through PI3K/AKT/mTOR axis in GC. Results present in Figure 4A-B demonstrated that overexpression of LHPP suppressed the phosphorylation of AKT, mTOR, and their downstream pathway molecules p70S6K(S371)/ p70S6K(T389), without affecting the total expression levels of AKT and mTOR. However, LHPP down-regulation increased the phosphorylation levels of AKT, mTOR, and p70S6K (S371)/ p70S6K (T389) (Figure 4C-D). Next, we used an mTOR inhibitor (AY-22989, 10nM) for the subsequent analysis. As shown in Figure 4E-G, CCK-8 and transwell assays demonstrated in LHPP-knockdown GC cells, the proliferation and migration abilities were markedly diminished by AY-22989 treatment. Those findings indicated that the LHPP downregulation promotes the malignant behavior of via the PI3K/AKT/mTOR pathway.

### The enzymatic active site exploration of LHPP

**Figure 5:**
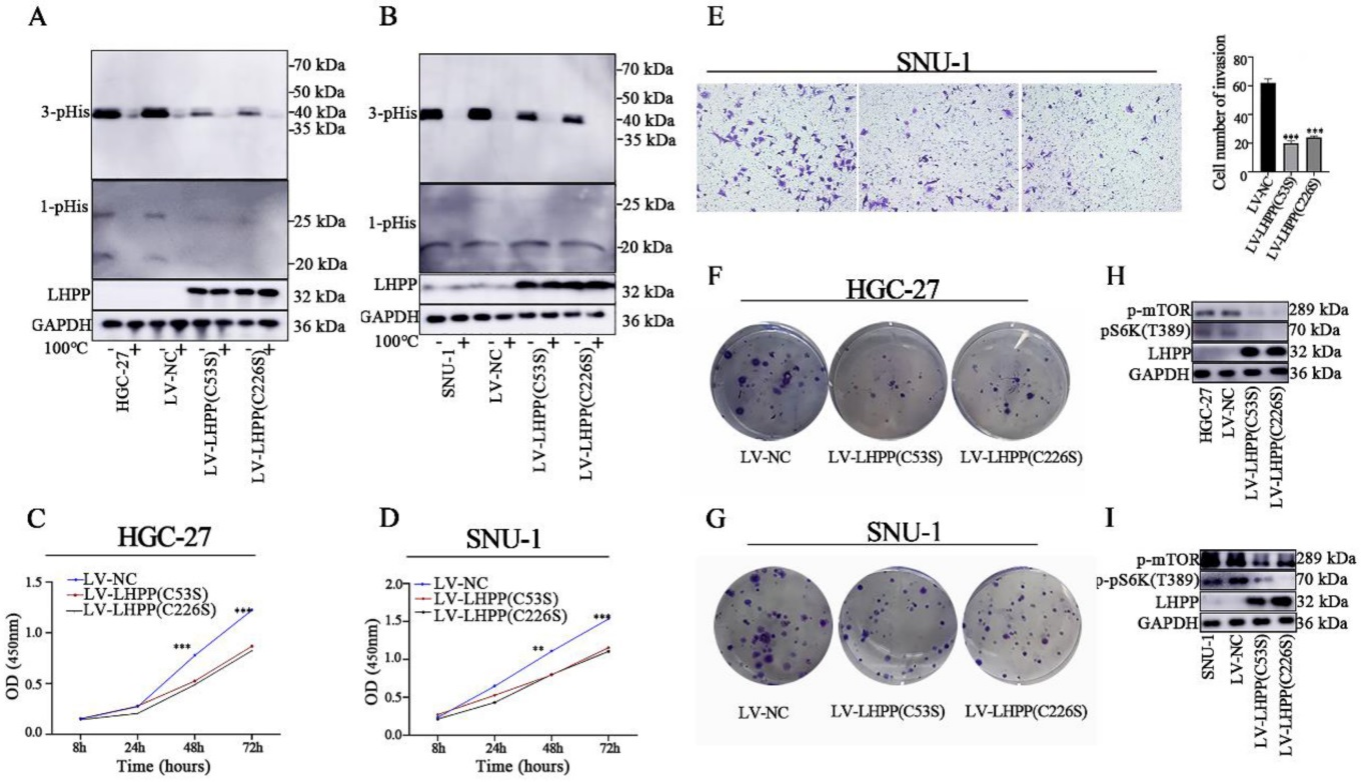
Western blotting analysis for intracellular 1-pHis and 3-pHis levels after overexpression of LHPP (C53S) mutant or LHPP (C226S) mutant in HGC-27 (A) and SNU-1 (B) cells. (C-D) Cell proliferative ability was detected by CCK-8 assay after overexpression of LHPP (C53S) mutant or LHPP (C226S) mutant in HGC-27 and SNU-1 cells. (E) Migration ability was assayed by transwell assay after overexpression of LHPP (C53S) mutant or LHPP (C226S) mutant in SNU-1 cells. (F-G) Cell colony formation capability was assayed by colony formation assay after overexpression of LHPP (C53S) mutant or LHPP (C226S) mutant in HGC-27 and SNU-1 cells. (H-I) Western blotting analysis for p-mTOR and p-pS6K(T389) levels after overexpression of LHPP (C53S) mutant or LHPP (C226S) mutant in HGC-27and SNU-1 cells.

It has been reported that dysregulated histidine phosphorylation participates in oncogenic. In this case, we decided to explore the protein histidine phosphatase ablilty of LHPP in GC cells by using western blot analysis. It was presumed that the specific enzymatic active site of LHPP is the cysteine residues at positions 53 and 226 2. We then constructed site-mutant LHPP proteins (C53S and C226S) and transfected them in GC cell lines, respectively. For histidine phosphorylation is sensitive to heat and acids, we divided each protein sample into two parts and used one as an internal control. Those control samples were then heated at 100℃ for 10min to remove phosphohistidine before SDS-PAGE to insuring the reliability of our results. As shown in Figure 5A-B, 3-pHis-positive proteins were well developed while 1-pHis proteins were hard to detect in both HGC-27 and SNU-1 cells. This may be caused by the thermodynamic stability of 1-pHis is lower than that of 3-pHis, or the phosphorylated proteins at N1 position is fewer than that at N3. Besides, compared with control groups, the intracellular histidine phosphorylation status was decreased in both the lv-LHPP (C53S) group and the lv-LHPP (C226S) group, indicating the catalytic effect does not been disrupted by point mutation. Moreover, neither the an-oncogenic effect of LHPP nor the PI3K/AKT/mTOR pathway was not been disrupted with LHPP site mutation (Figure 5C-G). Inconsistent with previous articles 2, 17, our data revealed that the enzymatic active site of LHPP may not be present in the cysteine residues at positions 53 and 226.

## Discussion

LHPP is a mammalian histidine phosphatases, catalyzing His-phosphorylated protein substrates dephosphorylation. It also involved in cell signal and metabolism. Until recently, researchers found LHPP function as a tumor suppressor gene in multiple cancers. Nevertheless, only one article concerns the relationship between LHPP and GC progression for now^12^. From our perspective, LHPP may exert its anti-tumor effects through multiple signaling pathways. Thus, we decided to further explore LHPP function in GC.

To investigate its expression pattern, we first analyzed LHPP mRNA expression in GC cell lines, the TCGA database, and tissue samples collected in our medical center. LHPP was found to pronouncedly decrease in tumor samples compared to normal tissues. Inconsistent with publicly available data, our clinicopathological parameters also showed that high LHPP expression indicates favorable OS. This discrepancy may due to different sample size or confounding bias. Therefore, further exploration is still needed to clarify the prognostic role of LHPP in GC.

For mechanism study, representative cell lines (SNU-1 and HGC-27) were stably transfected with OE-LHPP or shRNA-LHPP lentiviruses. Subsequent functional assays indicated that LHPP suppression stimulated cell proliferation and colony formation abilities *in vitro*, whereas overexpression of LHPP exhibited the opposite effects. Based on the findings above, we speculate that LHPP may exert anti-oncogenic effects in GC.

EMT is generally considered as a critical mechanism involved in tumor progression and metastasis. During this process, tumor cells gradually acquired mesenchymal properties, therefore promoting their dissemination to distant organs 18-20. EMT is characterized by epithelial-mesenchymal marker alteration and transcription factors activation/deactivation. For example, Snail induces EMT by inhibiting the CDH1 gene that encodes E-cadherin 21, 22. ZEB1 could regulate the expression of E-cadherin by directly bind to its promoter region 23. In this study, by conducting western blot analysis, we found that LHPP overexpression inhibited EMT. On the contrary, silencing of LHPP induced converse results. Consist with Lin et al. 12 our further experiments revealed that LHPP was critically involved in the regulation of the migration and invasion of GC cells. In the light of these findings, we concluded that LHPP might inhibit the migration, invasion abilities and EMT process of GC cells.

Accumulating evidence showed that the PI3K/AKT/mTOR pathway regulates various cellular processes 24-26. Moreover, several genes were reported to influence GC cell proliferation, invasion, and tumor angiogenesis via PI3K/AKT/mTOR signaling pathway 27, 28. Our previous study confirmed that LHPP has a strong connection with the PI3K/AKT signaling pathway in multiple tumors (unpublished). In the current study, our findings revealed that downregulation of LHPP in CC cell lines activated the PI3K/AKT/mTOR pathway. An mTOR inhibitor, AY-22989, attenuated the activation of the PI3K/AKT/mTOR pathway and suppressed the tumor malignant capabilities induced by LHPP knockdown. Consist with previous articles 7, 9, 16, our findings further confirmed the anti-tumor role of LHPP in GC, and may provide new insight into the mechanisms by which LHPP regulates GC cells progression.

As a unique phosphoamino acid, pHis consists of two isomers, namely 1-pHis or 3-pHis. Due to its nature of instability, the process of how pHis modifies proteins is still poorly characterized 29, 30. Until recently, technical difficulties were circumvented by the development of 1-pHis or 3-pHis monoclonal antibodies 30. Moreover, some researchers indicated that dysregulated histidine phosphorylation is associated with tumorigenesis and self-renewal ability of embryonic stem cells 6, 17. Thus, we decided to detect histidine phosphorylation levels following LHPP expression alteration in GC cells. Consistent with previous reports 17, fewer 1-pHis proteins were detected compared with 3-pHis proteins. However, our data indicated that the enzymatic active site of LHPP is neither the cysteine residue at position 226 nor at position 53 in GC. In summary, the enzyme function of LHPP in GC needs further investigation.

Our study also has some limitations. Firstly, the prognostic role of LHPP in GC is still controversial. More prospective, randomized, multicenter studies are still needed. Secondly, because of the COVID-19 pandemic, we re-scheduled our research plan and the *in vivo* validation is still needed. Thirdly, it is necessary to research deeply on more detailed mechanism analysis of LHPP in GC.

In summary, our study demonstrated that LHPP suppressed proliferation, migration, invasion and tumor formation of GC cells via regulation of the EMT *in vitro*, and the mechanism may be related to regulation of the PI3K/AKT/mTOR pathway. The above findings indicate that LHPP plays vital roles in the development and progression of GC and may be a novel therapeutic target.

## Supplementary Material

Supplementary figure and table.

## Figures and Tables

**Table 1:** Association between LHPP expression and clinicopathological characteristics of patients with stomach adenocarcinoma.

Parameters	Number of cases	LHPP expression		*P* value
		Low	High	
Age				
<60	18	9	9	0.41^ a^
≥60	34	21	13	
Gender				
Male	37	21	16	0.83^ a^
Female	15	9	6	
Pathological differentiation				
Well+moderate	23	10	13	0.07^ a^
Poor+undifferentiation	29	20	9	
Depth of tumor invasion				
T1+T2	14	8	6	0.96^ a^
T3+T4	38	22	16	
Lymph node metastasis				
Present	21	10	11	0.23^ a^
Absent	31	20	11	
TNM Stage				
Ⅰ, Ⅱ	20	14	6	0.16^ a^
Ⅲ, Ⅳ	32	16	16	

^a^ Usingχ2 test for this statistic.
