# Supplementary material for: LHPP suppresses gastric cancer progression via the PI3K/AKT/mTOR signaling pathway
Source: J Cancer. 2022 Oct 31;13(14):3584–92. doi: 10.7150/jca.78098 (PMC9723988; doi:10.7150/jca.78098)
Supplement: Supplementary file 1 — Supplementary figure and table. [file jcav13p3584s1.pdf]

Table S1. Comparison of patient baseline characteristics recruited from the TCGA project and our medical center.

Parameters	Dataset		*P* value
	TCGA	Local	
Age			
＜60	119	18	0.636[a]
≥60	261	34	
Gender			
Male	243	37	0.355 [a]
Female	137	15	
Pathological differentiation			
Well+moderate	141	23	0.361 [a]
Poor+ undifferentiation	239	29	
Depth of tumor invasion			
T1+T2	94	14	0.734 [a]
T3+T4	286	38	
Lymph node metastasis			
Present	120	21	0.21 [a]
Absent	260	31	
TNM Stage			
I，II	171	20	0.457 [a]
III，IV	209	32	

[a] Using $\chi 2$ test for this statistic

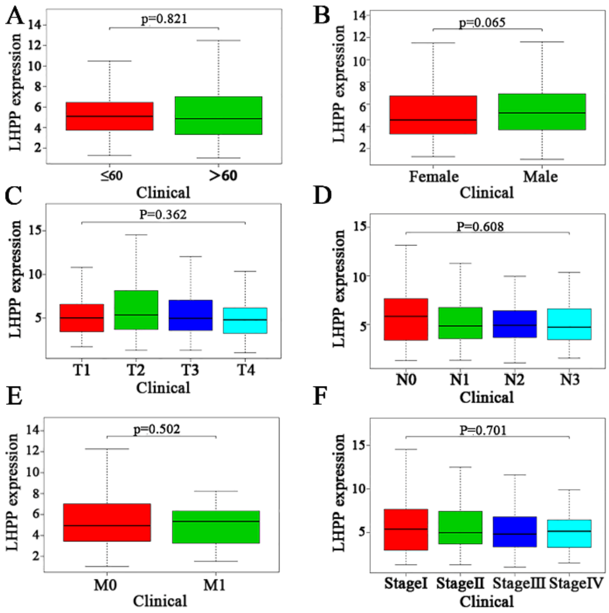

Figure S1. The relationship between the LHPP expression and clinicopathologic parameters (TCGA dataset). (A) Relative expression of LHPP in younger patients (≤60) or elder patients (>60). (B) Relative expression of LHPP in female or male patients. (C) Relative expression of LHPP in T stage 1, 2, 3, or 4. (D) Relative expression of LHPP in N stage 0, 1, 2, or 3. (E) Relative expression of LHPP in M stage 0 or 1. (F) Relative expression of LHPP in pathologic stage I, II, III, or IV
